# The Metabolic Impact of Nonalcoholic Fatty Liver Disease on Cognitive Dysfunction: A Comprehensive Clinical and Pathophysiological Review

**DOI:** 10.3390/ijms25063337

**Published:** 2024-03-15

**Authors:** Mauro Giuffrè, Nicola Merli, Maura Pugliatti, Rita Moretti

**Affiliations:** 1 Department of Internal Medicine (Digestive Diseases), Yale School of Medicine, New Haven, CT 06511, USA; 2 Department of Neuroscience and Rehabilitation, University of Ferrara, 44124 Ferrara, Italy; nicola.merli@unife.it (N.M.); maura.pugliatti@unife.it (M.P.); 3 Interdepartmental Research Center for Multiple Sclerosis and Other Inflammatory and Degenerative Disorders of the Nervous System, University of Ferrara, 44124 Ferrara, Italy; 4 Department of Clinical, Medical and Surgical Sciences, University of Trieste, 34149 Trieste, Italy

**Keywords:** NAFLD, NASH, insulin resistance, diabetes, gut microbiota, inflammation, gut–liver–brain axis, vitamins, free fatty acids, dementia, cognitive impairment, Alzheimer’s disease, neurodegeneration, hyperammonemia, vitamin A, vitamin B, vitamin D

## Abstract

Nonalcoholic fatty liver disease (NAFLD) exponentially affects the global healthcare burden, and it is currently gaining increasing interest in relation to its potential impact on central nervous system (CNS) diseases, especially concerning cognitive deterioration and dementias. Overall, scientific research nowadays extends to different levels, exploring NAFLD’s putative proinflammatory mechanism of such dysmetabolic conditions, spreading out from the liver to a multisystemic involvement. The aim of this review is to analyze the most recent scientific literature on cognitive involvement in NAFLD, as well as understand its underlying potential background processes, i.e., neuroinflammation, the role of microbiota in the brain–liver–gut axis, hyperammonemia neurotoxicity, insulin resistance, free fatty acids, and vitamins.

## 1. Introduction

Nonalcoholic fatty liver disease (NAFLD) is defined as a spectrum of diseases related to excessive fat deposition in the liver, ranging from simple steatosis (i.e., nonalcoholic fatty liver, NAFLD) to nonalcoholic steatohepatitis (NASH), characterized by lobular inflammation and hepatocyte ballooning, which over time can increase liver fibrosis, thus promoting liver cirrhosis or hepatocellular carcinoma [1,2,3]. The estimated global prevalence of NAFLD is approximately 25% in Western countries [4], with a high prevalence among young individuals, as shown by a recent UK study reporting a prevalence of 20% in people aged 22–26 years [4]. Despite the significant NAFLD disease burden, most of the affected individuals remain undetected, with less than 1% of individuals with NAFLD aware of their condition [5]. The progression from NAFLD to NASH is initially affected by fatty acids accumulation, which increases hepatocytes susceptibility to oxidative stress, insulin resistance, and proinflammatory cytokines, which, in the long term, promote steatohepatitis, chronic inflammation, and liver fibrosis. NAFLD occurs in the absence of secondary causes (e.g., alcohol abuse) and is associated with the presence of common cardiovascular risk factors (i.e., hypertension, obesity, visceral fat accumulation, and insulin resistance) [6,7,8,9]. Recognizing this close correlation to individual metabolic status, a recent consensus has suggested a change in nomenclature towards steatotic liver disease (SLD), which may help to identify those patients with NAFLD with poorer prognosis and increased risk for progression [10].

These metabolic risk factors associated with NAFLD are also known to be related to extrahepatic manifestations involving the central nervous system (CNS), including cognitive impairment and dementia (Table 1) [11,12,13,14,15,16,17,18,19,20,21,22]. A connection between NAFLD and impaired cognition has been proposed due to frequent reports of attention, forgetfulness, and memory issues in patients with NAFLD [23,24,25]. However, studies investigating the link between NAFLD and cognitive function have primarily focused on mild cognitive impairment using a cross-sectional approach [26,27,28]. In addition, the primary hypothesis connecting NAFLD to impaired cognition involves disruption in vascular-related pathways, as suggested by a higher prevalence of white-matter lesions in patients with NAFLD [27,28,29].

Despite the growing body of evidence linking NAFLD with cognitive impairment and dementia, the underlying mechanisms driving this association remain unclear. This gap in knowledge underscores the need for a more thorough exploration of the complex interplay between NAFLD and neurological outcomes. The aim of the current comprehensive narrative review is to delve deeper into this topic. We aim to not only summarize the existing clinical findings but also to investigate the underlying pathophysiological and pathobiological mechanisms that could link NAFLD to cognitive impairment and/or dementia.

## 2. Clinical Evidence

Several studies have explored cognitive performance in patients with NAFLD in the past two decades. A recent systematic review by George et al. [2] that included 11 studies with 7978 participants observed that individuals with NAFLD had poor cognitive performance in general cognition and specific cognitive domains [2]. However, due to the selection and inclusion criteria of this systematic review, the authors may not have included studies with contrasting results. Therefore, we have decided to summarize the main findings of the relevant literature, embracing reviews and observational and cross-sectional studies.

### 2.1. Evidence in Support of Correlation between NAFLD and Cognitive Impairment

As reported in Table 2, the collective findings of 21 studies indicate that NAFLD is associated with an increased risk of cognitive impairment and dementia, encompassing both Alzheimer’s disease and vascular forms of dementia [23,26,28,30,31,32,33,34,35,36,37,38,39,40,41,42,43,44]. Cognitive test scores were frequently lower among individuals with NAFLD, revealing a significant propensity for dysfunctions in frontal functioning and executive tasks, such as visuospatial perception, attention, concentration, mental speed, and reactivity, and also in ideation, abstraction, mental flexibility, learning, and working memory [2,26,28,31,33,35,36]. Language and verbal fluency appear less involved, although there are limited and more conflicting data [2,26,36].

Structural brain changes such as reduced brain and grey-matter volume, hippocampal volume loss, and altered functional and brain connectivity were associated with NAFLD [45,46,47]. Additionally, the severity of NAFLD, especially when combined with metabolic conditions like diabetes, was linked to more pronounced cognitive decline, chronic cerebrovascular dysfunction, and hypoperfusion [40]. Inflammatory markers, notably interleukin-6 (IL-6), were elevated in NAFLD patients and may contribute to the observed cognitive decline [41,48].

### 2.2. Evidence in Support of Correlation between NAFLD and Alzheimer’s Disease

Recent studies have suggested a potential connection between NAFLD and Alzheimer’s Dementia (AD), possibly related to shared genetic factors and similar disease mechanisms that influence both conditions [49]. Animal studies showed increased Aβ-plaque deposition in NAFLD models by decreased expression of low-density lipoprotein receptor-related protein 1 (LRP1) [50]. To support this hypothesis, a population-based study by Nho et al. [51], including 1581 participants with a mean age of 70 years, showed that patients with AD and NAFLD with elevated serum transaminases had poorer cognitive performances for memory and executive composite scores, increased Aβ-plaques deposition, and higher levels of phosphorylated tau/total tau protein in the cerebrospinal fluid (CSF) [51]. In addition, Weinstein et al. [52] studied a group of 169 NAFLD subjects performing β-amyloid and tau positron emission tomography (PET-CT), revealing a possible correlation between liver fibrosis (FIB-4) and early AD neuropathology, but without finding a clear association between β-amyloid or tau PET and prevalent NAFLD. Lastly, a systematic review and meta-analysis, including five cohort studies and two case-control studies published between 2020 and 2022 reported a significant increase in the risk of developing AD rather than vascular forms of dementia [53].

### 2.3. Evidence in Support of Correlation between NAFLD and Vascular Dementia

Vascular dementia, the second most common type of dementia, is primarily caused by vascular injuries, high blood pressure, reduced blood flow to brain tissues, high blood sugar, increased permeability of the blood–brain barrier (BBB), widespread inflammation in the body, and changes in gut bacteria [54]. Recent studies have explored the role of IL-6, a molecule involved in inflammation, in linking NAFLD to vascular dementia [41]. These findings suggest that the systemic inflammation seen in NAFLD may increase the risk of vascular dementia. Indeed, individuals with NAFLD are more likely to develop any form of dementia, particularly vascular dementia, compared to healthy individuals [44,55] or those with mild NAFLD [56]. In addition, NAFLD is closely associated with metabolic conditions like type 2 diabetes mellitus (T2DM), hypertension, and atherosclerosis. Patients with these conditions often experience reduced blood flow and vascular problems, such as endothelial dysfunction and vascular injury. These issues can lead to subclinical vascular injuries, impaired cerebrovascular reactivity, and changes in vascular function, similar to those observed in patients with vascular dementia [44,57].

### 2.4. Evidence against Correlation between NAFLD and Cognitive Impairment/Dementia

On the contrary, several investigations (Table 3) revealed that while initial observations suggested a link between NAFLD and reduced cognitive performance, the strength of these associations often diminished or disappeared after adjusting for cardiovascular and metabolic risk factors [58,59]. Some authors observed that patients with NASH developed mild cognitive impairment, thus presenting a minimal hepatic encephalopathy, while cognitive functions were not affected in patients with only NAFLD [60]. Other studies found that specific markers of liver disease, such as fibrosis, could enhance the prediction of cognitive decline, although NAFLD alone was not a consistent indicator of dementia risk [61,62]. Furthermore, it was noted that the presence of NAFLD did not consistently correlate with an increased risk for dementia or worse cognitive function in fully adjusted models. In some cases, NAFLD was even associated with a decreased risk for incident dementia within specific time frames after diagnosis [63]. Additionally, factors such as hyperammonemia and inflammation contributed to cognitive impairment only when they occurred together, suggesting a complex interplay between metabolic disturbances and brain health [60]. Regarding cognitive function across different populations, no significant associations were found between NAFLD and cognitive impairment, regardless of BMI, disease severity, or metabolic comorbidities. Instead, cognitive decline was more closely related to other factors, such as gender and the use of psychoactive medications [64]. Long-term studies also indicated no significant association between NAFLD and the risks of all-cause dementia, Alzheimer’s disease, or vascular dementia, further complicating the understanding of NAFLD’s role in cognitive health [65].

The current research on the relationship between NAFLD and cognitive impairment presents a complex and sometimes conflicting picture. The variability in research findings highlights the need for a more in-depth understanding of this association. In the following sections, we will explore the molecular and metabolic mechanisms underlying this relationship, focusing on how these factors might contribute to cognitive changes in individuals with NAFLD.

**Table 2:** The table summarizes findings from various clinical studies examining the relationship between nonalcoholic fatty liver disease (NAFLD) and cognitive performance, including risk factors and specific cognitive domains affected. It includes diverse study types ranging from cross-sectional to longitudinal cohort studies, with patient samples from different geographical locations and demographic backgrounds. Various cognitive tests were employed across studies to assess cognitive functions, and findings suggest a variable impact of NAFLD on cognitive impairment or dementia.

Authors	Location	Study Type and Patient Sample	Test Used	Findings Summary
**Cognitive Impairment**				
Liu et al. [30]	China	Longitudinal Study. 1651 middle-aged and elderly participants (>40 years) without cognitive impairment	MMSE	48.2% with NAFLD had higher 4-year incidence of cognitive impairment and 1.45-fold risk for cognitive impairment with NAFLD.
Weinstein et al. [26]	United States	Longitudinal Study. 1102 individuals identified from 19,931 NHANES participants. Of those eligible, 49.6% did not have NAFLD or T2DM, 21.7% had NAFLD only, 12.9% had T2DM only, and 15.8% had both NAFLD and T2DM.	MMSE AFT DSST	Individuals with both NAFLD and T2DM scored significantly lower on cognitive assessments than those with neither condition. For the AFT, those with T2DM only and those with both NAFLD and T2DM scored lower than those with neither condition (NAFLD only: 18.57 ± 0.31; T2DM only: 15.91 ± 0.54; both: 16.84 ± 0.45). For the DSST, similar results were found with lower scores for those with T2DM only and those with both NAFLD and T2DM compared to those with neither (NAFLD only: 55.99 ± 1.05; T2DM only: 45.08 ± 1.98; both: 47.12 ± 1.65).
Seo et al. [31]	United States	Cross-Sectional Study. 4472 adults aged 20–59 years who participated in NHANES III, with 874 fulfilling the ultrasound definition of NAFLD	SRTT, SDST, SDLT	NAFLD was independently associated with lower cognitive performance. Participants with NAFLD showed lower performance on the SDLT after controlling for demographic factors, cardiovascular disease, and cardiovascular risk factors. The associations with SRTT and SDST did not reach statistical significance after adjusting for such comorbidities.
Filipović et al. [32]	Serbia	Cross-Sectional Study. 89 first diagnosed therapy-naive patients with high levels of aminotransferases were involved, out of which 40 (22 men and 18 women) aged 34–57 years satisfied the recruiting criteria.	MoCA	Patients with NAFLD had lower cognitive statuses according to the MoCA index compared to controls. Specifically, 26 patients in the NAFLD group had lower MoCA scores than the cutoff value of 26 points, versus 6 control members. Patients with NAFLD had a significantly increased risk of cognitive impairment, with an odds ratio of 0.096 and a relative risk (RR) of 3.9 for cognitive impairment. Additionally, NAFLD significantly influenced cognitive deficits, correlating with white and grey-matter volume reduction, and patients with NAFLD were about four times more likely to have cognitive impairment.
Celikbilek et al. [33]	Turkey	Cross-Sectional Study. 70 participants with NAFLD and 73 age-matched and sex-matched healthy participants ranging from 18 to 70 years old.	MoCA	The MoCA scores were significantly lower in participants with NAFLD than in the healthy group. More NAFLD participants presented with deficits in the visuospatial and executive function domains. Education level and area of residence were independently associated with cognitive dysfunction in both the NAFLD and healthy groups.
Tuttolomondo et al. [42]	Italy	Cross-Sectional Study. 80 consecutive patients with biopsy-proven NAFLD and 83 controls without fatty liver disease.	MMSE	AFLD patients had a lower mean MMSE score compared to patients without NAFLD. NAFLD subjects in comparison to controls had (1) lower mean values of Reactive Hyperemia Index (RHI); (2) higher mean values of pulse wave velocity (PWV) and augmentation index (Aix); (3) lower MMSE scores; (4) a significant relationship at multivariate analysis between RHI and MMSE with NAFLD; (5) a significant negative relationship between ballooning grade and MMSE grade only in NASH subjects; and (6) a significant positive relationship between steatosis and augmentation index only in NASH subjects
Elliott et al. [23]	United Kingdom	Longitudinal Cohort Study. The study included consecutive patients attending the Newcastle Tertiary Liver Clinic with a histological diagnosis of NAFLD. In total, 224 NAFLD patients and 107 alcoholic liver disease (ALD) patients returned the assessment tools for analysis.	The study utilized validated functional, cognitive, autonomic, and fatigue symptom assessment tools completed by the patients.	Both NAFLD and ALD patients demonstrated significant functional difficulties that were worse than those of the control group. The functional impairment affected activities of daily living and persisted over the 3-year follow-up period. No significant difference in functional difficulty was observed between precirrhotic and cirrhotic participants.
Takahashi et al. [43]	Japan	Cross-Sectional Study. 24 female NAFLD patients without psychiatric disorders, including depression, and 15 age-adjusted healthy females	NIRS	NAFLD subjects had lower mean oxygenated-Hb concentration at baseline and during the fluency task, especially in the frontal lobes, and decreased brain activity reflected by poor cerebral oxygen reactivity and reduced word production.
Kang et al. [36]	South Korea	Cross-Sectional Study. The study included 4400 participants, of which 1415 (32.2%) had NAFLD based on the Fatty Liver Index (FLI) ≥ 30.	MMSE	Among the patients with NAFL, 666 (15.1%) participants had Mini-Mental Status Examination (MMSE) scores of <24, which was defined as cognitive impairment. Significant correlation between NAFLD (detected with Fatty Liver Index) and cognitive impairment after adjusting for socio-demographics, lifestyle factors, and comorbidities (OR = 1.26; 95% CI = 1.04–1.52) and in sensitivity analysis.
Yu et al. [35]	USA	Cross-Sectional Study. 4973 participants aged 20–59 from NHANES.	SDLT, SRTT, SDST	Subjects with NAFLD and metabolic dysfunctions (MD), but not NAFLD subjects without MD, were significantly predisposed to develop cognitive impairment, particularly in visual–motor speed tasks and response time. MAFLD independently associated with cognitive function, highlighting NAFLD’s systemic impact on lipid metabolism and chronic low-grade inflammation.
Cushman et al. [34]	USA	Nested Case-Control Study. 17,630 participants; 495 cases, 587 controls.	Six-item screener, animal fluency, word list learning, and recall	Significant association between cognitive impairment and NAFLD, suggesting NAFLD is a key risk factor for cognitive dysfunction.
Weinstein et al. [46]	United States	Cross-Sectional Study. 5660 NAFLD subjects with brain MRI.	Brain MRI	Detected lower total brain and grey-matter volumes in NAFLD subjects, significant even after demographic and clinical factors correction. Supporting the possible association between liver disease and brain aging.
Miao et al. [45]	China	Cross-Sectional Study. 225 participants, 70 with fMRI.	fMRI, cognitive tests	Significant correlation between NAFLD and poor memory performance, hippocampal volume loss, and functional brain-imaging alterations in NAFLD subjects.
Xu et al. [47]	China	Cross-Sectional Study. 44 NAFLD subjects, 20 healthy controls.	MoCA, DST, TMT-A, resting-state fMRI	NAFLD subjects had inferior cognitive performance, and nonobese NAFLD patients had abnormal functional connectivity in various brain regions.
An et al. [48]	USA	Cross-sectional cohort of 23 NAFLD patients	RBANS (Repeatable Battery for the Assessment of Neuropsychological Status, exploring immediate and delayed memory, attention, language, and visuospatial memory)	Association between high levels of CTRP13 (tumor necrosis factor-related proteins involved in metabolic regulations and obesity) and poor cognitive performance, especially in visuospatial memory, in NAFLD subjects.
Weinstein et al. [28]	United States	Cross-Sectional (Framingham Heart Study). 1287 participants (mean age = 61 ± 12 years, 48% men), of whom 378 (29%) had nonalcoholic fatty liver disease (NAFLD).	Standardized neuropsychological test battery including the Wechsler Memory Scale, Trail-making Test, Similarities test (SIM), and the Hooper Visual Organization Test (HVOT)	The presence of NAFLD was not associated with cognitive function. However, NAFLD with high risk for advanced fibrosis was associated with poorer performance on the similarities test and trail-making test (specifically the difference in time between Trail-making B and A), indicating a potential link between liver fibrosis severity and reduced cognitive function in executive function and abstract reasoning.
Weinstein et al. [44]	United States	Cross-Sectional Study (Framingham Heart Study). 766 participants (53.5% women, mean age = 67 years), of whom 137 (17.9%) had nonalcoholic fatty liver disease (NAFLD).	Brain MRI measures include total cerebral brain volume, hippocampal and white matter hyperintensity volumes, and presence or absence of covert brain infarcts.	NAFLD was significantly associated with smaller total cerebral brain volume (β [SE] −0.26 [0.11]; *p* = 0.02), corresponding to an accelerated brain aging of 4.2 years in the general sample and up to 7.3 years in individuals younger than 60 years. No significant associations were found between NAFLD and other MRI measures like hippocampal volume, white matter hyperintensity volume, or covert brain infarcts.
**Dementia**				
Shang et al. [37]	Sweden	Longitudinal Cohort Study. 2898 patients with NAFLD aged 65 y or older; 28,357 controls.	Not Reported	Significant association between NAFLD and dementia, with an HR of 1.86 for developing dementia in NAFLD patients, which weakened after adjustment for comorbidities. Enhanced association if cardiovascular comorbidities.
Jeong et al. [38]	Korea	Longitudinal Study. 608,994 adults aged ≥60 years.	Not Reported	8% developed dementia and 7.7% developed Alzheimer’s disease (AD), with a significant association between NAFLD and increased risk of incident dementia, particularly AD.
Weinstein et al. [52]	FHS	Cross-Sectional Study. Based on a subsample of 169 NAFLD subjects from the Framingham Heart Study.	β-amiloyd or tau deposition on PET	Prevalent NAFLD was not associated with β-amyloid or tau onPET. FIB-4 was significantly associated with increased rhinal tau (β = 1.03 ± 0.33, *p* = 0.002). FIB-4 was related to inferior temporal, parahippocampal gyrus, entorhinal, and rhinal tau and to β-amyloid deposition overall and in the inferior temporal and parahippocampal regions.
Solfrizzi et al. [39]	Italy	Longitudinal Study. 1061 older adults with NAFLD aged 65 to 84 years. The focus was on assessing the risk of dementia over an 8-year follow-up period.	Not Reported	Higher fibrosis score in NAFLD patients was associated with an increased overall risk of dementia, HR of 4.23.
Moretti et al. [40]	Italy	Cross-Sectional Study. 319 adult patients diagnosed with subcortical vascular dementia (sVaD).	Frontal Assessment Battery (FAB), Hamilton Anxiety Rating Scale (HAM-A), Apathy Evaluation Score (AES-C), Neuropsychiatric Inventory (NPI), and Quality of Life in Dementia Scale (QUALID). Additionally, clinical and biochemical parameters, including liver enzymes and ultrasound for NAFLD assessment, were utilized.	Patients with comorbid NAFLD and sVaD had worse neuropsychological outcomes, including higher scores in HAM-A (indicating anxiety), AES-C (indicating apathy), and NPI (indicating neuropsychiatric symptoms), and a lower FAB score (indicating poorer executive function). These patients also had a worse metabolic profile, characterized by higher levels of homocysteine and deficiencies in vitamins B12, D, and folate.
Wang et al. [41]	China	Population-based cross-sectional study including 5129 participants (aged ≥60 years; 61.79% women) living in rural communities.	Diagnosis of dementia, Alzheimer’s disease (AD), and vascular dementia (VaD) following international criteria.	Out of 5129 participants, 455 (8.87%) had moderate-to-severe NAFLD. Dementia was diagnosed in 292 participants (5.69%), including 188 with AD and 96 with VaD. The study found that moderate-to-severe NAFLD (compared to no-to-mild NAFLD) had odds ratios of 2.22 for all-cause dementia, 1.88 for AD, and 2.62 for VaD. Additionally, in the cytokine subsample, moderate-to-severe NAFLD was significantly associated with higher levels of certain serum cytokines, such as interleukin-6, which mediated 12.56% of the association between NAFLD and VaD.

Abbreviations List—NAFLD: Nonalcoholic Fatty Liver Disease; MMSE: Minimental State Examination; T2DM: type 2 diabetes mellitus; AFT: Auditory Verbal Learning Test; DSST: Digit Symbol Substitution Test; SRTT: Simple Reaction Time Test; SDST: Symbol Digit Substitution Test; SDLT: Serial Digit Learning Test; MoCA: Montreal Cognitive Assessment; NIRS: near-infrared spectroscopy; FLI: Fatty Liver Index; FAB: Frontal Assessment Battery; HAM-A: Hamilton Anxiety Rating Scale; AES-C: Apathy Evaluation Score; NPI: Neuropsychiatric Inventory; QUALID: Quality of Life in Dementia Scale; NHANES: National Health and Nutrition Examination Survey; MRI: magnetic resonance imaging; fMRI: functional magnetic resonance imaging; DST: Digit Symbol Test; TMT-A: Trail Making Test Part A; RHI: Reactive Hyperemia Index; PWV: Pulse Wave Velocity; Aix: Augmentation Index; ALD: alcoholic liver disease; sVaD: subcortical vascular dementia; OR: odds ratio; CI: confidence interval; FHS: Framingham Heart Study; RS: Rotterdam Study.

**Table 3:** This table summarizes research findings on the association between NAFLD and cognitive impairment, including dementia, from a range of international studies. The included studies utilize various research designs, such as longitudinal cohort studies and cross-sectional analyses, with diverse patient populations, including middle-aged adults, individuals with diabetes or hypertension, and those undergoing evaluation for bariatric surgery. Cognitive function was assessed using a battery of tests, including standard cognitive assessments and neuroimaging techniques. The findings collectively indicate a complex relationship between NAFLD and cognitive function, with some studies showing no significant association after adjusting for confounding factors, while others report specific cognitive domains being affected in NAFLD patients.

Authors	Location	Study Type and Patient Sample	Test Used	Findings
Gerber et al. [58]	United States	Longitudinal Cohort Study (CARDIA). Participants included 2809 middle-aged adults (average age 50.1 years, 57% female, 48% black) with CT examination and cognitive assessment at Year 25 (Y25, 2010–2011) and reassessed at Year 30 (Y30, 2015–2016).	DDST, RAVLT	The study found that NAFLD was inversely associated with cognitive scores at baseline, but after adjustment for cardiovascular disease (CVD) risk factors, no associations were shown between NAFLD and cognitive scores. Similarly, no associations were observed with 5-year cognitive decline. The study concluded that inverse associations between NAFLD and cognitive scores among middle-aged adults were attenuated after adjustment for CVD risk factors, with the latter being predictive of poorer cognitive performance both at baseline and follow-up.
Shang et al. [61]	Sweden	Retrospective matched cohort study. 656 patients with biopsy-proven NAFLD and 6436 matched controls from the general population.	Dementia incidence was ascertained using National registers	There was no significant association between NAFLD and the incidence of dementia. However, adding histological markers, particularly fibrosis stage, to a conventional risk model for dementia did enhance its predictive capacity. This suggests a potential shared metabolic origin between NAFLD and dementia.
Basu et al. [59]	United States	Post hoc analyses of data from two randomized controlled trials (ACCORD and SPRINT studies). The ACCORD trial included 2969 diabetic participants with a mean age of 62 years, and the SPRINT trial included 2890 hypertensive participants with a mean age of 68 years.	Cognitive function tests (Mini-Mental Status Examination, Digital Symbol Substitution Test, Stroop Color–Word Test, Rey Auditory Verbal Learning Test) and brain magnetic resonance imaging volume measurements.	The study found no consistent associations between liver disease and cognitive performance or brain volumes at baseline or longitudinally after adjustment in both the ACCORD and SPRINT populations. The study concluded that markers of chronic liver disease were not associated with cognitive impairment or related brain-imaging markers among individuals with diabetes and hypertension.
Xiao et al. [63]	Netherlands	Longitudinal and cross-sectional analyses within the Rotterdam Study. Participants included three different sets: Set 1 (3975 participants aged 70 years, follow-up 15.5 years), Set 2 (4577 participants aged 69.9 years, follow-up 5.7 years), and Set 3 (3300 participants aged 67.6 years, follow-up 5.6 years).	Dementia diagnosis according to the DSM-III-R criteria, cognitive function assessment using neuropsychological tests (Stroop test, Letter Digit Substitution Test, Word Fluency Test, 15-Word Learning Test, and Purdue Pegboard Test).	NAFLD and fibrosis were not consistently associated with an increased risk for dementia or worse cognitive function in fully adjusted models. Interestingly, NAFLD was associated with a significantly decreased risk for incident dementia until 5 years after Fatty Liver Index assessment. The study concluded that NAFLD and fibrosis were not linked to increased dementia risk, nor was NAFLD associated with impaired cognitive function.
Felipo et al. [64]	Spain	The study included patients with different liver or dermatological diseases, assessing the presence of mild cognitive impairment. The groups included patients with liver cirrhosis (n = 35), NAFLD (n = 11), NASH (n = 11), psoriasis (n = 20), and keloids (n = 22).	PHES [comprises the Digit Symbol Test (DST), the Number Connection Test A and B (NCT-A and NCT-B), the Serial Dotting Test (SD), and the Line Tracing Test (LTT)]	The study found that 5/11 of patients with NASH had mild cognitive impairment (MCI), thus presenting a minimal hepatic encephalopathy, while cognitive functions were not affected in patients with only NAFLD Hyperammonemia or inflammation alone did not induce cognitive impairment. However, the combination of certain levels of hyperammonemia and inflammation was enough to induce cognitive impairment, even in the absence of liver disease.
Wernberg et al. [64]	Denmark	Cross-sectional study. The study included 180 patients undergoing evaluation for bariatric surgery with a body mass index of 35 kg/m^2^. Of these, 72% were women, the average age was 46 ± 12 years, 78% had NAFLD, and 30% had NASH without cirrhosis.	Continuous Reaction Time Test, Portosystemic Encephalopathy Syndrome Test, and the Stroop Test. A representative subgroup also underwent RBANS. The triggering receptor expressed on myeloid cells 2 (TREM2) was used as a biomarker for neuronal damage.	8% of the patients were cognitively impaired according to the basic tests, and 41% showed cognitive impairment based on RBANS results. The most affected cognitive functions were executive and short-term memory. The study found no associations between cognitive impairment and BMI, NAFLD presence or severity, or metabolic comorbidities. However, male sex and the use of two or more psychoactive medications were associated with cognitive impairment. TREM2 levels were not associated with cognitive impairment.
Huang et al. [56]	United Kingdom	Prospective analysis of 179,222 UK Biobank participants. NAFLD was diagnosed based on the Fatty Liver Index.	Cox proportional hazards models were used to estimate the adjusted hazard ratio (HR) and 95% confidence interval (CI) for incidence.	During a median follow-up of 12.4 years, 4950 incident dementia cases were identified, including 2318 Alzheimer’s disease (AD) cases and 1135 vascular dementia (VD) cases. There was no significant association between NAFLD and the risks of all-cause dementia (HR: 0.97, 95% CI: 0.90–1.06), AD (HR: 0.95, 95% CI: 0.84–1.07), or VD (HR: 1.03, 95% CI: 0.88–1.22). The meta-analysis of prospective studies, which included 879,749 subjects, also found no significant association between NAFLD and incident dementia, with a pooled HR for all-cause dementia of 1.01 (95% CI: 0.94–1.08) and for VD of 0.99 (95% CI: 0.86–1.13). The study concluded that there was no evidence of an association between NAFLD and incident dementia.
Labenz et al. [62]	Germany	Population-based cohort study. Elderly patients (≥65 years) with NAFLD, total of 44,634 patients (22,317 with NAFLD and 22,317 without NAFLD) from 1262 general practices in Germany.	Analysis based on ICD-10 coding in the Disease Analyzer Database. Primary outcomes were all-cause dementia diagnoses, incidence of vascular dementia, and antidementive drug prescription.	Over 10 years, 16.0% of patients with NAFLD and 15.6% without NAFLD were diagnosed with dementia. The study found no association between NAFLD and the incidence of all-cause dementia (HR 0.97, 95% CI 0.92–1.04), vascular dementia (HR 0.89, 95% CI 0.78–1.02), or the new prescription of antidementive therapy (HR 0.87, 95% CI 0.76–1.01).

Abbreviation List—CARDIA: coronary artery risk development in young adults; DDST: Digit Symbol Substitution Test; RAVLT: Rey Auditory Verbal Learning Test; PHES: Psychometric Hepatic Encephalopathy Score; RBANS: Repeatable Battery for the Assessment of Neuropsychological Status; TREM2: triggering receptor expressed on myeloid cells 2; BMI: body mass index; NASH: nonalcoholic steatohepatitis; HR: hazard ratio; CI: confidence interval; AD: Alzheimer’s Disease; VD: Vascular Dementia; ICD-10: International Classification of Diseases, Tenth Revision; DSM-III-R: Diagnostic and Statistical Manual of Mental Disorders, Third Edition, Revised.

## 3. Molecular Evidence

### 3.1. Insulin Resistance and Diabetes-Promoted Neurodegeneration

Insulin is a peptide hormone secreted by the β-pancreatic cells and regulates energy metabolism by controlling glucose uptake in the liver, fat, and muscle cells by interaction with its receptors also located in several brain regions, such as the cerebral cortex, hippocampus, and hypothalamus [66]. Insulin resistance, defined as reduced insulin sensitivity and detected in approximately 40% of patients with NAFLD [67], arises from the reduced expression of insulin receptors, ineffective insulin-receptor bindings, or impairment in insulin signaling [66,68]. In the brain, activating phosphoinositide 3-kinase (PI3K)/Akt signaling, mediated by insulin/insulin growth factor I (IGF-I) binding to its receptor, affects axon development and synaptic formation. Impaired insulin action contributes to altered glucose metabolism, neuronal damage, and impaired secretion of neurotransmitters in the brain [69,70]. In particular, a study in animal models showed that animals with insulin resistance in NAFLD had lower brain-glucose levels and metabolism impairment, which was also observed in individuals with AD [71].

Also, insulin can influence hippocampal synaptic plasticity and memory formation through the involvement of the long-term potentiation process. In fact, brain insulin resistance is known to lead to memory loss, impaired learning, and early development of dementia [72]. In addition, brain insulin signaling results in tau hyperphosphorylation by activating the glycogen synthase kinase 3 (GSK-3β) [73].

Insulin resistance is one of the main risk factors promoting the onset of diabetes, particularly T2DM, with affected individuals showing NAFLD prevalence of 55.5% [74]. Hyperglycemia affects the distribution of tight-junction (TJ) proteins and nutrient transporters across the BBB, altering its permeability and function as shown in in vitro models [75] and magnetic resonance imaging (MRI) in patients with T2DM [76]. The CNS effects of insulin resistance and diabetes were shown to be more marked in individuals with the apolipoprotein E alleles *ε4* and *ApoE-ε4* allele, which was found to be present in 16.6% of patients with NALFD [77].

In addition, impaired levels of adiponectin (ADPN), a plasma protein with anti-inflammatory and neuroprotective activity, have been related not only to obesity and diabetes and worsening insulin resistance but have also been observed in MCI, AD, and vascular dementia [78].

### 3.2. Systemic Inflammation

Systemic inflammation is common in patients with NAFLD, which can be considered a chronic inflammatory disease [79]. Supporting evidence comes from the Framingham Heart Study in 2019; after multivariate correction for main cardiovascular risk factors, NAFLD patients showed significantly higher serum concentrations of inflammatory biomarkers, including high-sensitivity C-reactive protein (hs-CRP), intercellular adhesion molecule 1 (ICAM-1), P-selectin, interleukin 6 (IL-6) and urinary isoprostanes [80]. Hepatocytes actively secrete hepatokines, which are proteins that can influence metabolic processes through autocrine, paracrine, and endocrine signaling [81,82,83], such as Fetuin-A, often elevated in NAFLD [84], which was found to promote endothelial and vascular dysfunction in NALFD via tol-like receptor-4 (TLR-4) and tumor necrosis factor-α (TNF-α) [82]. In addition, TNF-α overexpression was observed in the liver and adipose tissue of patients with metabolic syndrome [85], being related to obesity and insulin resistance. TNF-α has proinflammatory properties through ROS production and transcriptional pathways, mainly relying on NF-kB and *mitogen-activated protein kinase* (*MAPK*) mediators [86]. Endothelial TNF-α activation leads to the overexpression of adhesion molecules (such as ICAM-1 and VCAM-1) and increased vascular permeability [87,88]. NAFLD and NASH patients also have higher serum interleukin-6 (IL-6) and soluble IL-6 receptor α levels than healthy controls [89]. IL-6 has three signaling modalities, referred to as “trans-signaling” (where IL-6 uses the soluble form of the IL-6Rα as a receptor), activation via JAK/STAT intracellular mediators, leading to hepatic and systemic inflammatory responses, endothelial proinflammatory changes [87,90,91,92] and inducing insulin resistance, acute liver damage, regeneration, and chronic liver inflammation [92]. Recent evidence shows that IL-6 may have a possible disease-limiting role in liver inflammation and remodeling through the induction of miR-223 secretion by myeloid cells [93], insulin resistance, and adipose tissue macrophage accumulation in animal models [92]. IL-1-type cytokines are either secreted by immune cells and epithelial cells, also by the liver and adipose tissue, or activated via cleavage by neutrophil proteases in response to endogenous damage biomarkers known as damage-associated molecular patterns (DAMPs) or pathogen-associated molecular patterns (PAMPs) translocated from the gut microbiome [94]. After ligand binding on several cell types of targets, through the activation of NF-κB and MAPK, IL-1 receptors trigger proinflammatory, prothrombotic responses and the secretion of other cytokines [83,84,85] and are also implied in insulin resistance, adipose tissue inflammation, and atherosclerosis [95]. MAPK-pathway activation in leukocytes leads to increased avidity of cell-surface integrins for endothelial adhesion molecules (ICAMs and VCAMs), effectively promoting adhesion and extravasation of immune cells [96].

In NAFLD, activated immune cells contribute to the production and secretion of several proinflammatory cytokines, leading to endothelial and pericyte activation and changes in BBB permeability, thus favoring immune-cell extravasation and M1-like (or proinflammatory) activation of local microglia, producing what is known as neuroinflammation [97,98]. Direct cytokine-mediated neurodegeneration also plays a role in neurotoxicity, with in vivo evidence for IL-1, IL-6, and TNF-α [99,100,101,102,103]. Moreover, overexpression of the inducible isoform of nitric oxide synthase (iNOS) by activated macrophages and microglia leads to nitric oxide production, which modulates and increases neurotoxicity in concert with other cytokines expression [99]. This process damages neuronal axons and myelin sheaths in a propagating vicious cycle sustained by cytokine production, such as TNF-α, IL-1β, and IL-6 [97,98].

On a larger scale, this neurovascular unit (as intended, the functional and anatomical joint between neurons, astrocytes, and microvessels regulating neural activity and blood flow) uncoupling has been associated with changes in local cerebral perfusion and BBB integrity, with accumulation of toxic products of cerebral metabolism. Chronic neuroinflammation by the activated microglia and cytokines is observed in the brains of patients with AD and T2DM [104,105]. Thereby, chronic or systemic inflammation promotes the secretion of inflammatory molecules that further worsen NAFLD and promote neuroinflammation.

### 3.3. Neuroinflammation

Neuroinflammation seems to be also promoted by the migration of cytokines, chemokines, and other inflammatory mediators from systemic circulation through the blood–brain barrier (BBB), yielding microglial activation and inflammation spreading within the central nervous system (CNS). Furthermore, the essential role of metabolic syndrome has to be taken into account, where obesity, type II diabetes, and hypertension are well-established risk factors for vascular forms of cognitive deterioration.

In support of this proinflammatory theory, Fiorillo et al. [106] emphasized the putative involvement of the immune system, analyzing leukocyte populations by flow cytometry and plasma cytokine levels of 71 NAFLD patients and 61 healthy controls, finding increased levels of CD69 in CD4+ T cells, activation of Th17 lymphocytes, increased IL-17 production, and many other cytokines in patients with mild cognitive impairment, plausibly leading to immune dysfunction and potentially facilitating BBB disruption and infiltration of immune cells and inflammatory mediators. Interestingly, recent experimental research based on animal models conducted by Hadjihambi et al. [107] observed that genetically modified mice are haploinsufficient for monocarboxylate transporter-1 (*MCT1*)—a ubiquitous cotransporter of short-chain fatty acids, lactate, and ketone bodies with a proinflammatory role also involved in microglial activation—show a NAFLD-resistant phenotype despite obesogenic diet and adipose tissue accumulation, protecting from liver, cerebrovascular, glial and proinflammatory systemic alterations. By contrast, animals with a complete expression of MCT1 experienced hepatic steatosis, low-grade brain-tissue hypoxia, and inflammation supported by microglial and astrocytic morphological and metabolic dysfunctions, decreased cerebral cortex vessel density, and behavioral changes [108].

### 3.4. Gut–Liver–Brain Axis

The human gastrointestinal system is inhabited by a large group of 2000 distinct species of bacteria (approximately 100 trillion bacteria), in addition to archaea, fungi, microbial eukaryotes, and viruses, that exist in a symbiotic relationship with each other and their human host [109]. The adult gut is primarily inhabited by five bacterial phyla: Firmicutes (79.4%), Bacteroidetes (16.9%), Actinobacteria (2.5%), Proteobacteria (1%), and Verrumicrobia (0.1%) [109]. The gut–liver–brain axis is a multidirectional interaction system regulating homeostasis between the CNS, liver, and the gut involving the neural, endocrine, and immune systems [89,110,111,112]. Lately, growing interest is being attributed to the gut microbiota and its relationship with different neurological conditions such as stroke, involvement in pathogenesis, potentially improving or worsening the prognosis of cerebrovascular events [113], mediating immunomodulatory therapy’s effects in multiple sclerosis [114], and contributions to AD pathogenesis and progression [115].

Related to cognitive function, rising scientific evidence confirms a reasonable microbiota involvement. Cattaneo et al. [116,117] studied a cohort of patients with cognitive impairment, finding a decrease in levels of anti-inflammatory bacteria and an abundance of proinflammatory species in subjects with cognitive dysfunction and brain amyloidosis at PET. Consistently, Vogt et al. [117] analyzed the gut microbial composition in individuals with and without AD, detecting a lowering of microbial richness and variety in AD patients vs. controls, hypothesizing the correlation between the brain–gut axis and pathophysiological alterations of CNS.

Disruption of the microbial equilibrium is often seen in NAFLD patients, especially considering that the type of dysbiosis (e.g., increased Firmicutes–Bacteroides ratio, the relative abundance of Proteobacteria, Enterobacteriaceae, Escherichia, and Dorea vs. decreased abundances of Ruminococcaceae, Anaerospacter, Coprococcus, Eubacterium, Faecalibacterium, and Prevotella) affecting these patients alters intestinal permeability (tight-junction dysfunction), promotes bacterial translocation, and chronic inflammation (increased permeability to bacterial products and endotoxins), increments the production of ammonia in the gut, and oxidative stress [89,118]. In addition, gut microbiota could influence lipid metabolism via the upregulation of lipoprotein lipase, which is involved in the release of fatty acids, thus promoting increased cellular uptake of fatty acids and adipocyte triglycerides accumulation [119]. This mechanism directly promotes liver steatosis and insulin resistance [119]. In this framework, the role of the liver–gut axis and its intercommunication becomes essential, as the hepatic system constantly receives catabolites, metabolites, and microbiota elements from portal vein blood and concomitantly transfers bile into the small intestine. Hence, high fat/high sugar diet, drugs, and microbiota products, such as fatty acids and bile acids, can concur to generate microbiota imbalance, altering the vagal–gut pathway and the liver’s glycolipid homeostasis, promoting gut inflammation, compromising intestinal permeability, and, therefore, contribute to NAFLD development. According to this pathogenetic model, a possible interaction between microbiota and mitochondria activity has been described since microbiota dysbiosis could dysregulate the mitochondria’s consumption of fatty acids and bile acids, the primary substrate for energy production and biogenesis [56,120]. Thus, this process may lead to mitochondrial dysfunction with consequent reactive oxygen species (ROS) accumulation and increased hepatic oxidative stress.

Regarding scientific evidence relating NAFLD dysbiosis and cognitive impairment, Higarza et al. [121] found that animal NAFLD models had lower levels of short-chain fatty acids in the gut, together with depressive-like behavior, short-term memory impairment characterized by deficits in social recognition and spatial working memory [121]. These findings were also related to lower metabolic brain activity in the prefrontal cortex, thalamus, and hippocampus, and decreased dopamine release [121], which showed a benefit from short-period probiotic intervention [122]. Another study by Li et al. realized a randomized Mendelian analysis of 211 gut microbiota samples, observing that specific taxa of bacteria appeared to be associated with NAFLD progression and maintenance [123].

### 3.5. Hyperammonemia-Induced Neurotoxicity

Ammonia production occurs in all human body tissues due to the metabolism of nitrogen-containing compounds. Ammonia is metabolized by the urea cycle, located exclusively in the liver, which is the main process for eliminating ammonia. Recent findings have shown that NAFLD is associated with impaired liver function, even in the early stages of the disease. In particular, it has been shown that liver fat accumulation is associated with a reduction in mitochondrial gene expression of protein related to the urea cycle, leading to a reduced functional capacity of urea synthesis, even at the stage of simple steatosis, thus leading to increased accumulation of ammonia, even at early stages of the disease. To further support this hypothesis, the functional capacity of the urea cycle was found to be restored to normality after dietary interventions and a decrease in liver fat.

In physiological conditions, blood concentrations of ammonia must remain very low, because even slightly elevated concentrations were found to be neurotoxic [124,125]. The positive charge of the ionic form NH4^+^ prevents its passage across the BBB so that only the gaseous form (NH_3_), which represents only a small fraction of the whole-body ammonia, accesses the brain through diffusion [126]. However, as the increase of intrahepatic fat accumulation progresses, damaged hepatocytes secrete excess proinflammatory cytokines that can activate the microglia, resulting in increased permeability of BBB [127], thus allowing increased permeability to NH4^+^ ions. In the brain, ammonia is metabolized into glutamine by the glutamine synthetase after glutamate binding [126]. The elevation of CSN ammonia concentration induces an increase in glutamate in astrocytes, which has a synaptic effect of excitotoxic damage combined with calcium overload and oxidative stress [128]. At the same time, the increased ammonia metabolism in astrocytes results in glutamine accumulation, which leads to osmolyte-inducing astrocytic swelling and metabolism disruption, leading to cerebral edema [129].

### 3.6. Free Fatty Acids

Free fatty acids (FFA) are carboxylic acids with an aliphatic chain, which is either saturated or unsaturated, which serve as important energy substrates and are released by the hydrolysis of triglycerides within the adipose tissue by lipoprotein lipase [130]. FFAs are a risk factor for T2DM, NAFLD, and cardiovascular disease. Diet and nutrient patterns modulate cerebral lipid composition and brain functions [131]. Among FFAs, N-3 and N-6 polyunsaturated fatty acids (PUFAs) regulate vasoreactivity and participate in the modulation of neuroinflammation [132,133]. Monounsaturated fatty acids (MUFAs) have been recently studied for their possible impact on the brain; intake of MUFAs promotes insulin action in the brain with its beneficial effects on cortical activity, locomotion, and sleep [133]. Recently, Pinçon et al. [131] reported that, in a mouse AD model, diet-induced NAFLD contributes to the imbalance of brain cholesterol and MUFA and N-3 PUFA deficiency. These events promote neuroinflammation and favor oxidative stress by increasing the general brain hypoperfusion state and altering Aβ-peptide levels in the bloodstream [131]. Also, further experiments showed that the removal of a high-fat diet decreased the Aβ-plaque load in transgenic mice and reversed signs of systemic and cerebral inflammation [50,134]. In contrast, the animals who maintained a high-fat diet for twelve months were characterized by a rapid increase in the number and dimensions of Aβ-plaques, which are associated with a progressive neural and glial cell death, general cortical shrinkage, tau-protein hyperphosphorylation, increase of neurofibrillary tangles, and severe signs of cerebral amyloid angiopathy. Wild-type mice developed significant signs of neuroinflammation, with a comparable upregulation of TNF-α, IL-1β, IL-6, IL-7, TLR-1, TLR-2, and TLR-6 gene expression, which usually recognize lipoproteins and glycolipids [135].

### 3.7. Vitamin Deficiency

Vitamin deficiency has been associated with NAFLD development and progression [136].

Vitamin A is a fat-soluble micronutrient with retinoic acid being its active metabolite [137]. The role of vitamin A has not been extensively explored in patients with NAFLD [138,139,140]. However, individuals with NAFLD show significantly lower levels of vitamin A when compared to healthy controls, especially those with a higher degree of liver fibrosis [138,139,140]. Vitamin A is primarily stored in quiescent hepatic stellate cells, which, when activated for fibrogenesis, secrete their vitamin A reservoir, which becomes lost in the process [138,139,140]. Vitamin A is critical in the CNS, involved in early brain development, function, and synaptogenesis [141]. Vitamin A receptors were found to be genetically linked to AD [142], given the fact that the activation of vitamin A receptors can downregulate the expression of β-secretase enzymes [137]. Hypovitaminosis A or receptor dysfunction has been associated with increased Aβ-plaques deposition and impairment in hippocampal synaptic plasticity [137].

The B vitamins comprise a group of eight water-soluble vitamins that perform essential and inter-related functions in cellular metabolism, acting as coenzymes in several catabolic and anabolic enzymatic reactions, including mitochondrial energetic production and the folate–methionine cycles [143]. The importance of B vitamins for physiological brain functions resides in the fact that (1) each vitamin is actively transported to the brain across the BBB; (2) brain B-vitamin concentrations reach 50 times the concentration detected in blood; and (3) the high brain turnover of B vitamins range from 8% to 100% daily [143,144,145]. Only a few of the eight B vitamins have been investigated in patients with NAFLD.

Vitamin B3 (niacin), is present in two active forms, nicotinic acid and nicotinamide, and acts as a precursor for the coenzyme nicotinamide adenine dinucleotide (NAD) and nicotinamide adenine dinucleotide phosphate (NADPH). With the progression of liver disease and increasing age [146], the liver NAD pool is consumed by the accumulation of DNA breaks that activate poly ADP-ribose polymerases (PARPs) [147]. NAD deficiency reduces β-oxidation rates, thus favoring the accumulation of triglycerides in hepatic cells and increasing oxidative stress, insulin resistance, and liver inflammation [148]. In the brain, vitamin B3 promotes neuronal survival and maintains endothelial vascular integrity, and its deficiency has been linked to AD [149]. In fact, NAD depletion and mitochondrial dysfunction have usually been found at AD onset [150,151]. Also, vitamin B3 counteracts amyloid-induced toxicity by reducing the expression of the amyloid precursor protein gene and by reducing the production of reactive oxygen species (ROS) [152,153].

The functions of vitamin B9 (folate or folic acid) and vitamin B12 (cobalamin) are inextricably linked to each other to their complementary roles in the folate and methionine cycles. Higher rates of deficiency of both Vitamin B9 and B12 have been detected in patients with NALFD, particularly in those with more severe disease [154], and in patients with dementia [155,156]. Vitamin B9 shows hepatoprotective effects due to its ability to restore adenosine monophosphate-activated protein kinase (AMPK) activation, whose deficiency has been associated with liver steatosis, insulin resistance, and hyperglycemia [157]. Vitamin B12, which is primarily stored in the liver, influences DNA synthesis/repair and mitochondrial metabolism, whose damage is commonly implicated in NAFLD pathogenesis [139,158] and, in the brain, can alter neuronal differentiation/repair, induce hippocampal atrophy, demyelination, and impair the integrity of neuronal membranes [159]. In addition, vitamin B12 deficiency results in a so-called “functional folate deficiency” because folate becomes trapped in the form of methyl tetrahydrofolate [159,160]. Altered pathways in the folate cycle reduce the production of tetrahydrobiopterin, which is a key cofactor from several enzymes that convert amino acids to neurotransmitters and nitric oxide, thus altering neural transmission [161,162].

Vitamin D consists of a group of fat-soluble secosteroids primarily responsible for calcium, magnesium, and phosphate metabolism. Dietary vitamin D must be converted into its bioactive form, 1,25-dihydroxyvitamin D, by 25-hydroxylase and 1α-hydroxylase. In the liver, low levels of Vitamin D activate TLRs, promoting inflammation and oxidative stress. Also, the expression of Vitamin D receptors (VDR) decreases as the liver disease progresses, especially in the case of lobular inflammatory damage [163,164,165]. The activation of VDR in liver macrophages and hepatic stellate cells promotes the attenuation of hepatic inflammation and fibrosis and increases insulin sensitivity by intensifying the intracellular expression of intracellular insulin receptor substrate (IRS)-1 and insulin-dependent glucose transporters on fat tissue [164,166,167]. Both neurons and neuroglia are rich in VDR and 1-hydroxylase [163], which can interact and metabolize Vitamin D upon crossing the BBB. Once in the brain, the complex vitamin D–VDR can interact with the vitamin-A receptor and decrease the expression of β-secretase enzymes involved in Aβ-plaques formation [168]. Also, vitamin D affects microglial cells by increasing the expression of interleukine-10 (IL-10)-producing genes that normally show anti-inflammatory properties, thus preventing neuroinflammation [169,170].

## 4. Conclusions

**Figure 1:**
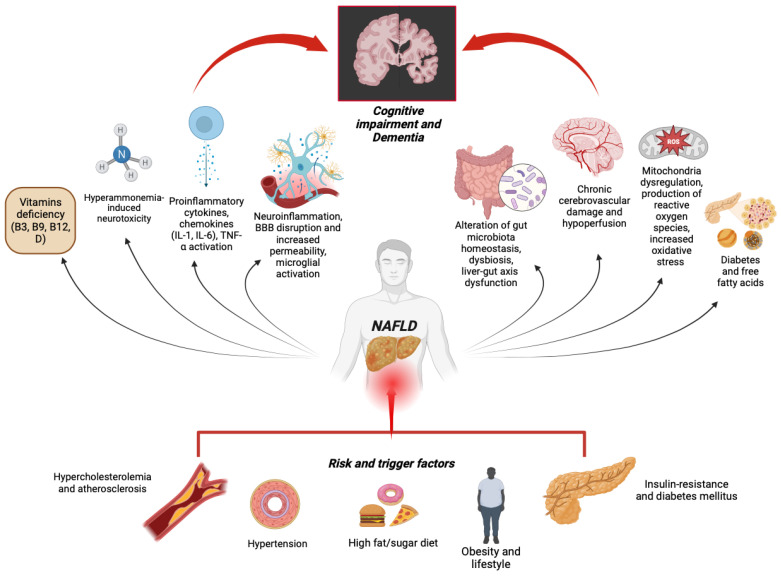
Insulin resistance is a critical factor in NAFLD, leading to disrupted brain insulin signaling and contributing to neurodegeneration, including Alzheimer’s disease (AD). The resistance impairs insulin/IGF-I mediated neuroprotective pathways, exacerbating cognitive decline. NAFLD patients, particularly those with T2DM and the ApoE-ε4 allele, experience altered BBB permeability, exacerbating CNS conditions. Systemic inflammation in NAFLD is characterized by high levels of proinflammatory biomarkers, contributing to neuroinflammation, endothelial dysfunction, and cerebral hypoperfusion, together with cardiovascular risk factors. The gut–liver–brain axis shows that gut dysbiosis in NAFLD impacts metabolic and cognitive functions, suggesting a link between microbiota and neurodegenerative diseases. Hyperammonemia due to NAFLD leads to neurotoxicity through increased BBB permeability and astrocytic swelling. Imbalances in FFA metabolism associated with NAFLD are risk factors for neurodegenerative and cardiovascular diseases, influencing brain health. Vitamin A, B, and D deficiencies in NAFLD patients impact brain development, function, and inflammation, linking to cognitive disorders and neurodegeneration. This integrated perspective highlights the importance of addressing metabolic dysfunctions to mitigate neurodegenerative diseases.

In this review, we summarized the current findings, elucidating the possible mechanisms behind NAFLD and cognitive impairment or dementia (Figure 1) and summarized the main findings of most of the studies, evaluating a possible correlation between NAFLD and impaired cognition/dementia, highlighting findings that both support and contrast this hypothesis. The altered metabolic status of individuals with NAFLD can directly affect brain activity, primarily by altering BBB integrity, thus allowing the transit of inflammatory or exogenous molecules, and leading to sustained neuroinflammation. In addition, gut dysbiosis plays a crucial role by altering the gut barrier and its interplay with the liver, allowing the entrance of bacterial products that can promote systemic inflammation. Moreover, several factors, including dietary vitamin deficiencies, can promote Aβ-plaques deposition, thus worsening AD outcomes. Certainly, under the reported data and the future increase of patients with NAFLD and dementia, future observational studies must embrace a well-defined evaluation and stratification of NAFLD risk factors, considering subjects’ comorbidities and ethnicity, and a wide-ranging thorough multidomain cognitive assessment. As dementia presents a deteriorative and irreversible course, preventive interventions are indispensable to lower this burden. It has been suggested that modifying potential risk factors for dementia might prevent or delay 40% of dementia [171]. Indeed, awareness and systematic screening of such a condition are crucial to establishing a prompt multidisciplinary approach to prevent and delay earlsy cognitive deterioration, ultimately aiming to develop supportive predictive diagnostic algorithms.

## Figures and Tables

**Table 1:** Diagnostic criteria according to DSM-5 of cognitive impairment and dementia reported with key prevalence characteristics worldwide.

Diagnostic Criteria and Key Characteristics	Prevalence
**Cognitive Impairment**	
Modest cognitive decline in one or more cognitive domains, based on: − Concern about mild decline, expressed by an individual or reliable informant or observed by the clinician; − Modest impairment, documented by objective cognitive assessment. No interference with independence in everyday activities, although these activities may require more time and effort, accommodation, or compensatory strategies. Cognitive impairment affects cognitive functions such as reasoning, perception, memory, verbal, mathematical, and problem-solving abilities and reduces the individual capability of performing more complex everyday tasks (e.g., housework, driving, and working).	Approximately 50 million individuals worldwide are affected by cognitive impairment, and it is estimated to increase to 2 billion individuals by 2050. The worldwide prevalence of mild cognitive impairment (MCI) among community inhabitants over 50 years of age is 15.56% according to a recent meta-analysis, and is affected by age, gender, education level, and region of study sites. A meta-analysis of 41 studies revealed a cumulative proportion of 39.2% of MCI cases who progressed to dementia in specialist settings. Moreover, around 22% in population studies develop a major cognitive disorder over the following 3 to 10 years, in contrast with 3% of the population without MCI.
**Dementia**	
Significant cognitive decline in one or more cognitive domains, based on: − Concern about significant decline, expressed by an individual or reliable informant or observed by the clinician; − Substantial impairment, documented by objective cognitive assessment. Interference with independence in everyday activities. Dementia can have different etiologies (vascular dementia, Alzheimer’s Disease, frontotemporal dementia, Lewy body disease) and is characterized by significant memory loss, confusion, personality changes, and difficulty in speaking, understanding, and expressing language, and reading and writing. Depending on the underlying etiology some forms may be characterized by visual hallucinations, Parkinsonian movement features, or even drastic changes in social behavior and personality.	Approximately 55 million individuals worldwide are affected by dementia, with Alzheimer’s Disease contributing to 60–70% of cases. Dementia is the seventh most common cause of death and a significant contributor to disability and dependency in the elderly worldwide. In 2019, dementia incurred a global economic cost of USD 1.3 trillion, with around 50% of these expenses being linked to care given by informal caregivers, such as family members and close friends, who typically offer 5 h of care and supervision daily. Women are more significantly impacted by dementia, both directly and indirectly. Women have a greater burden of disability-adjusted life years and mortality from dementia, while also contributing 70% of the caregiving hours for individuals with dementia.

## Data Availability

Not applicable.
